# Sociodemographic characteristics associated with frequency and duration of eating family meals: a cross-sectional analysis

**DOI:** 10.1186/s40064-016-3739-3

**Published:** 2016-12-01

**Authors:** Margie R. Skeer, Konstantina E. Yantsides, Misha Eliasziw, Migdalia R. Tracy, Allison R. Carlton-Smith, Anthony Spirito

**Affiliations:** 1 Department of Public Health and Community Medicine, Tufts University School of Medicine, 136 Harrison Avenue, Boston, MA 02111 USA; 2 Division of Clinical Psychology, Department of Psychiatry and Human Behavior, Alpert Medical School of Brown University, Box G-BH, Providence, RI 02912 USA

**Keywords:** Family meals, Sociodemographic characteristics, Parents, Children, Cultural differences

## Abstract

**Introduction:**

Children who frequently eat family meals are less likely to develop risk- and behavior-related outcomes, such as substance misuse, sexual risk, and obesity. Few studies have examined sociodemographic characteristics associated with both meal frequency (i.e., number of meals) and duration (i.e., number of minutes spent at mealtimes).

**Methods:**

We examine the association between sociodemographics and family meal frequency and duration among a sample of 85 parents in a large New England city that was recruited through the public-school system. Additionally, we examined differences in family meals by race/ethnicity and parental nativity. Unadjusted ANOVA and adjusted ANCOVA models were used to assess the associations between sociodemographic characteristics and frequency and duration of meals.

**Results:**

Sociodemographic characteristics were not significantly associated with the frequency of family meals; however, in the adjusted models, differences were associated with duration of meals. Parents who were born outside the U.S. spent an average of 135.0 min eating meals per day with their children compared to 76.2 for parents who were born in the U.S. (*p* < 0.01). Additionally, parents who reported being single, divorced, or separated on average, spent significantly more time per day eating family meals (126.7 min) compared to parents who reported being married or partnered (84.4; *p* = 0.02). Differences existed in meal duration by parental nativity and race/ethnicity, ranging from 63.7 min among multi-racial/other parents born in the U.S. to 182.8 min among black parents born outside the U.S.

**Discussion:**

This study builds a foundation for focused research into the mechanisms of family meals. Future longitudinal epidemiologic research on family meals may help to delineate targets for prevention of maladaptive behaviors, which could affect family-based practices, interventions, and policies.

## Background

In recent years, the lay community has promoted family meals as a way to prevent risk-related behaviors and outcomes, such as substance use and obesity (Skeer and Ballard 2013), among youth. According to a recent review of the literature (Skeer and Ballard 2013), observational studies have demonstrated that families who frequently eat meals together have closer relationships, and it is believed that such relationships reduce children’s likelihood of subsequently developing behavior-related problems, due to increased parent–child communication and improved social-interaction skills in children (Fiese et al. 2006). Family meals also provide an opportunity for parents to listen to their children talk about their daily lives (Fulkerson et al. 2010), thus strengthening their bonds (Griffin et al. 2000). This could lead to increased levels of confidence and trust between family members (Utter et al. 2013).

Given the potential benefits for children, it is important to understand what factors are related to the frequency and duration (i.e., the number of minutes spent) of family meals. However, research examining the sociodemographic characteristics associated with eating family meals has been scant. Separate studies conducted by Neumark-Sztainer and Larson, along with their colleagues, examined the association between sociodemographic profiles of families with adolescents and family meal frequency (Neumark-Sztainer et al. 2003, 2013; Larson et al. 2013), and they consistently found that boys, mothers who were not employed, those identifying as Asian American, and individuals with high socioeconomic status had family meals more frequently, compared to those of other sociodemographic characteristics (Neumark-Sztainer et al. 2003). However, to the best of our knowledge, no studies have examined sociodemographic factors associated with mealtime duration in any age group. Additionally, little research to date has examined the sociodemographic factors associated with all meals, including breakfast, lunch, and dinner; much of the work has focused solely on dinner. Because not all families can eat dinner together due to scheduling or activities, not capturing all meals could limit our understanding of how family meals, in total, could affect children’s risk outcomes. Furthermore, while myriad studies have examined the association between family meals and their effect on adolescent outcomes (Eisenberg et al. 2004, 2008; Fulkerson et al. 2008, 2010; Sen 2010), the vast majority of them have focused on the frequency of meals (i.e., the number of meals eaten together per week). However, there is a dearth of research on the total duration of time families spend together eating meals. If time spent together communicating and bonding is the mechanism by which family meals confer a protective effect, then in theory, duration of time eating meals together as a family would increase the dose of this protective effect. For example, families who eat three 60 min meals per week have two hours more time eating together per week than families who eat three 20 min meals. Understanding both the duration and frequency of meals, rather than frequency alone, will help to provide a comprehensive view of how family meals may be associated with a reduction in risk behaviors and outcomes among youth.

Another gap in the literature is that the majority of previous research has focused on the adolescent population and excludes elementary and middle school children. Such exclusion could be hindering research efforts, because the earlier in life children eat meals with their families, the stronger the effect on child developmental outcomes may be (Fulkerson et al. 2008). Finally, cultural variability exists in family meals. For example, certain countries emphasize meals as a time to socialize with family members, friends, and other community members, which contrasts patterns in the U.S. of eating alone or outside the home (Bevis 2012). The emphasis on family meals outside the U.S. could suggest that families from particular ethnic backgrounds and/or newly acculturated families eat more meals together and spend more time together eating at each meal compared to other ethnicities and/or acculturated families.

To expand upon the family meals research literature, we examined the sociodemographic characteristics associated with the number of meals eaten per week and the duration (i.e., number of minutes) of meals among a sample of parents in a major city in New England. Because research has shown that culture and background influence family meals (Ochs and Shohet 2006; Trends 2013), we specifically examined differences in family meals by race/ethnicity and parental nativity. We hypothesized that those born outside the U.S. would eat more meals with their children per week and for more minutes per day than those born in the U.S. This study contributes to a comprehensive view of family meals and builds a foundation so that more focused research into the mechanisms of family meals can be conducted.

## Methods

### Sample and recruitment

For this cross-sectional study, we analyzed baseline data that were collected as part of a family meals intervention trial. The data were collected from 86 parents of third through sixth grade students in five schools from one school district, who participated in the intervention between April 2013 and December 2014. The school district is diverse, with 40% identifying as Hispanic, 35% as black, and 13% as white. For recruitment, study staff attended open houses, after-school programs, and approached parents during school pickup. To participate, individuals had to: (1) be a parent or legal guardian of a child in third through sixth grades in the school system, (2) spend at least 5 days per week with the child, and (3) be able to speak English or Spanish well enough to complete study procedures. Parents of children with special needs were excluded from participation. All study procedures were approved by the university academic medical center Institutional Review Board and from the research oversight office of the school district where the research was conducted.

### Measures

Parents completed an hour-long survey that inquired about demographics, family meals, and the relationship with their child. The survey, which was available in English and Spanish, was administered through a Computer-Assisted Survey Instrument (CASI).

#### Frequency and duration of family meals

Family meals were defined for the participants as “a meal where you sat down with your child (the one in the study with you) and one of you was eating, regardless of what type of food was served.” We defined family meals this way because we hypothesized that it is not the act of eating that provided the potentially protective effect of family meals, rather it is the time that parents and children spend together. Therefore, it was less important that everyone at the meal ate something.

Family meals were measured through two methods: First, we employed an adapted timeline follow-back method (Robinson et al. 2014), in which parents were asked to report the number and duration of each breakfast, lunch, and dinner for every day over the past seven days that they ate with the child in the study, starting with the day before the survey administration. In a separate section, we inquired how many breakfasts, lunches, and dinners participants ate on average with their child during a typical week during the past month, and how long these meals typically lasted. The timeline follow-back questions and overall meal questions for each meal were strongly correlated (all with Pearson correlation coefficients *r* > 0.70), and were therefore averaged to create aggregate measures of mealtime frequency and mealtime duration for each meal. Finally, for both frequency and duration, we summed breakfast, lunch, and dinner to derive the following outcomes: (1) total number of family meals per week, and (2) total number of minutes spent eating meals together per day in an average week.

#### Gender and child grade

Data on parent and child gender, as well as child grade were collected. For the purpose of the analyses, child grade was dichotomized as grades three through four and five through six, which aligned with the stratified recruitment strategy for the study.

#### Race/ethnicity

Using NIH guidelines, participants were asked about their race and ethnicity separately. Ethnicity was assessed as Hispanic or non-Hispanic; for race, participants selected as many categories as applied from a list of race options and also had a space to write in additional designations. Because the majority of those who identified as Hispanic checked “other” for race or wrote variations on “Hispanic,” and due to small numbers of certain races, we combined race and ethnicity and analyzed them together as white, black, Hispanic, or other/multiracial.

#### Education

Respondents indicated their highest completed level of education, ranging from no high school degree through doctoral-level degree. Responses were dichotomized for analyses as: high school degree/GED or less versus greater than high school.

#### Income

Nine response options were available, ranging from less than $5000 through $150,000 or more. Participants were asked to indicate total household income from all members. For analyses, responses were categorized as: less than $25,000; between $25,000 and $74,999; and $75,000 or greater.

#### Parental nativity

Participants were asked if they were born in the United States, and data were analyzed as born in the U.S. versus not born in the U.S.

#### Marital status

Participants were asked to choose the answer that best characterized their marital status: married; living with partner/unmarried; single, never married; separated/divorced; or widowed. For analysis purposes, we categorized married and living with a partner together, and single and separated/divorced together. No participants were widowed.

#### Single caretaker family

We did not directly assess the number of caretakers in a household; rather, we constructed this variable using a question about the education level of other caretakers in the household. If in response to this question, participants answered “there is only one adult caretaker in the household,” they were categorized as a single-caretaker household. If they answered the question by indicating the education level of other caretakers, they were categorized as having multiple caretakers in the home. Having another caretaker in the home could be a spouse or another adult family member, such as a grandparent or aunt/uncle.

#### Siblings living in the household

Respondents indicated how many brothers and sisters their child lives with in the same household. For analyses, responses were categorizes as zero, one, two, and three or more.

### Data analysis

We calculated means and proportions to summarize the sociodemographic characteristics. For both meal frequency and meal duration, we assessed their association with each characteristic individually using a one-way analysis of variance, yielding unadjusted means. To account for potential confounders, we used analysis of covariance to re-assess the same associations by including all measured characteristics in the model (including age of parent, parent and child gender, child grade, race/ethnicity, parental nativity, parental marital status, number of caretakers in the household, number of child’s siblings in the household, parental education, and household income), yielding adjusted means. In an exploratory analysis, we examined the combined effect of race/ethnicity and parental nativity on family meals by adding a cross-product term to the analysis of covariance model. All analyses were conducted using SAS v. 9.3 (SAS Institute Inc, Cary, NC) and results with *p* < 0.05 were considered to be statistically significant.

## Results

Among the 86 parents who enrolled, one reported not having eaten any family meals with the child in the study during the data collection period of interest and has been excluded from the present analyses. The sociodemographic characteristics of the remaining 85 parents, who reported eating at least one family meal, are presented in Table 1. Participants were primarily female (90.6%), and approximately half of the families (55.3%) had daughters. The sample was racially/ethnically representative of the population within the school system and more than half (60.0%) were born in the U.S. Approximately one-third of the participants (30.6%) lived in a single-parent household and all of the participants identified as biological parents; there were no adoptive parents or other guardians in the study. The income distribution of participants was also representative of the population from which it was drawn, with close to one-third reporting an annual household income of less than $15,000 and approximately one-quarter above $50,000.

**Table 1:** Demographic characteristics of the sample

Age of parent in years; mean (SD)	37.7 (6.8)
Parent gender, n (%)	
Female	77 (90.6)
Male	8 (9.4)
Child gender, n (%)	
Female	47 (55.3)
Male	38 (44.7)
Child grade, n (%)	
3rd or 4th	48 (56.5)
5th or 6th	37 (43.5)
Race/ethnicity, n (%)*	
Hispanic/Latino/Latina/Spanish	32 (37.6)
Black	42 (49.4)
White	22 (25.9)
Asian	1 (1.2)
Multi-racial/other	23 (27.1)
Born in the U.S., n (%)	
Yes	51 (60.0)
No	34 (40.0)
Marital status, n (%)	
Married	28 (32.9)
Living with partner, unmarried	15 (17.7)
Single, never married	30 (35.3)
Separated/divorced	12 (14.1)
Number of caretakers in household, n (%)	
Single-parent	26 (30.6)
Multiple caretakers	59 (69.4)
Number of siblings in household, n (%)	
0	19 (22.3)
1	26 (30.6)
2	23 (27.1)
3 or more	17 (20.0)
Education, n (%)	
No high school	9 (10.6)
High school/GED	29 (34.1)
Some college/2 year degree	30 (35.3)
4 year degree	5 (5.9)
Graduate degree	12 (14.1)
Household income in U.S. dollars, n (%)	
≤14,999	27 (31.8)
15,000–49,999	33 (38.8)
≥50,000	20 (23.5)
Not reported	5 (5.9)
Number of meals per week; mean (SD)	
Total number	10.9 (3.8)
Breakfast	3.5 (2.0)
Lunch	2.0 (1.5)
Dinner	5.7 (1.7)
Number of minutes per meal; mean (SD)	
Total for all meals (n = 85)	99.6 (66.3)
Breakfast (n = 83)	33.9 (26.4)
Lunch (n = 76)	44.5 (30.9)
Dinner (n = 83)	50.5 (28.8)

* Race/ethnicity does not sum to 100% as respondents were able to select multiple options

Table 2 summarizes the associations by comparing the mean frequency and mean duration of the meals between the categories of each demographic characteristic. There were no associations with frequency of weekly meals as all the *p*-values were greater than 0.10. In regards to duration of meals, although there were apparent differences for a number of characteristics, parental nativity was the only statistically significant variable, where those who were born outside of the U.S. had a significantly greater (42.3 min more, *p* = 0.003) number of minutes of family mealtime per day compared to those born in the U.S.

**Table 2:** Comparison of mean number of meals per week and mean number of minutes in meals per day

Characteristic	Family meals			
	Mean number of meals per week (SD)	*p* value	Mean number of minutes per day (SD)	*p* value
Age of parent				
<40 years (n = 54)	10.6 (4.0)	0.38	104.4 (70.7)	0.38
40 years or older (n = 31)	11.3 (3.6)		91.2 (57.9)	
Parent gender				
Female (n = 77)	10.9 (3.8)	0.93	95.9 (53.0)	0.11
Male (n = 8)	10.8 (4.1)		135.1 (143.5)	
Child gender				
Female (n = 47)	10.8 (3.7)	0.91	108.5 (63.5)	0.17
Male (n = 38)	10.9 (4.0)		88.7 (68.8)	
Child grade				
3rd or 4th (n = 48)	11. 0 (4.0)	0.72	88.0 (38.8)	0.07
5th or 6th (n = 37)	10.7 (3.6)		114.7 (88.7)	
Race/ethnicity				
White (n = 13)	12.0 (3.2)	0.41	88.3 (72.5)	0.64
Black (n = 38)	10.1 (3.9)		108.2 (73.8)	
Hispanic (n = 24)	11.2 (4.0)		99.5 (61.8)	
Multi-racial/other (n = 10)	11.3 (3.8)		82.0 (31.6)	
Born in the U.S.				
Yes (n = 51)	10.4 (3.6)	0.20	82.7 (35.2)	0.003
No (n = 34)	11.5 (4.1)		125.0 (90.5)	
Marital status				
Single/separated/divorced (n = 42)	10.3 (4.0)	0.17	104.8 (71.3)	0.48
Married/living with partner (n = 43)	11.4 (3.6)		94.6 (61.4)	
Number of caretakers in household				
Single-parent (n = 26)	10.7 (4.4)	0.78	88.2 (42.1)	0.29
Multiple caretakers (n = 59)	10.9 (3.6)		104.7 (74.2)	
Number of siblings in household				
0 (n = 19)	10.8 (4.3)	0.40	93.9 (53.9)	0.96
1 (n = 26)	9.9 (3.9)		103.8 (96.4)	
2 (n = 23)	11.6 (3.8)		97.0 (41.6)	
3 or more (n = 17)	11.5 (3.1)		103.2 (52.8)	
Education				
High school/GED or less (n = 38)	10.8 (4.3)	0.82	94.7 (42.7)	0.54
Some college or more (n = 47)	11.0 (3.5)		103.6 (80.7)	
Household income in U.S. dollars				
≤14,999 (n = 27)	10.7 (3.6)	0.64	106.2 (61.4)	0.88
15,000–49,999 (n = 33)	10.4 (4.5)		98.1 (72.5)	
≥50,000 (n = 20)	11.7 (3.3)		97.7 (69.7)	
Not reported (n = 5)	11.4 (2.7)		81.5 (43.3)	

*SD* standard deviation

Accounting for potential confounders did not markedly change the associations with frequency of weekly meals (Table 3). None of the associations were statistically significant and the mean differences remained small. On the other hand, accounting for potential confounders identified three other significant associations with duration of family meals: male parents reported eating an average of 53.4 min more than female parents (*p* = 0.04); parents with daughters reported eating an average of 30.8 min longer per day than parents with sons (*p* = 0.04); and those who were single or separated/divorced ate 42.3 min more per day than those married or living with a partner (*p* = 0.02). Although not statistically significant, being a younger parent, identifying as black, having multiple caretakers in the home, and having a higher education was also associated with eating longer.

**Table 3:** Comparison of adjusted mean number of family meals per week and adjusted mean number of minutes in family meals per day from an analysis of covariance

Characteristic	Family meals			
	Mean number of meals per week (SE)	*p* value	Mean number of minutes per day (SE)	*p* value
Age of parent				
<40 years (n = 54)	12.0 (1.1)	0.73	120.3 (17.7)	0.07
40 years or older (n = 31)	12.4 (1.1)		90.9 (17.1)	
Parent gender				
Female (n = 77)	12.1 (0.8)	0.93	78.9 (11.5)	0.04
Male (n = 8)	12.2 (1.7)		132.3 (25.9)	
Child gender				
Female (n = 47)	11.6 (1.1)	0.22	121.0 (16.4)	0.04
Male (n = 38)	12.8 (1.2)		90.2 (17.6)	
Child grade				
3rd or 4th (n = 48)	12.1 (1.1)	0.85	96.4 (17.5)	0.21
5th or 6th (n = 37)	12.3 (1.1)		114.8 (16.5)	
Race/ethnicity				
White (n = 13)	13.2 (1.6)	0.63	97.0 (23.9)	0.11
Black (n = 38)	11.6 (1.2)		135.7 (18.4)	
Hispanic (n = 24)	11.2 (1.2)		98.8 (18.8)	
Multi-racial/other (n = 10)	12.7 (1.6)		90.9 (24.1)	
Born in the U.S.				
Yes (n = 51)	11.2 (1.0)	0.08	76.2 (15.8)	<0.001
No (n = 34)	13.1 (1.2)		135.0 (18.8)	
Marital status				
Single/separated/divorced (n = 42)	11.4 (1.1)	0.19	126.7 (17.0)	0.02
Married/living with partner (n = 43)	12.9 (1.2)		84.4 (18.3)	
Number of caretakers in household				
Single-parent (n = 26)	12.8 (1.4)	0.29	87.5 (21.0)	0.06
Multiple caretakers (n = 59)	11.5 (0.9)		123.7 (14.5)	
Number of siblings in household				
0 (n = 19)	12.0 (1.2)	0.08	100.8 (18.5)	0.47
1 (n = 26)	10.2 (1.2)		93.4 (17.8)	
2 (n = 23)	13.6 (1.4)		103.1 (21.4)	
3 or more (n = 17)	12.8 (1.4)		125.1 (21.4)	
Education				
High school/GED or less (n = 38)	12.5 (1.2)	0.46	91.5 (18.3)	0.07
Some college or more (n = 47)	11.8 (1.0)		119.7 (15.9)	
Household income in U.S. dollars				
≤14,999 (n = 27)	12.1 (1.2)	0.55	109.1 (17.6)	0.73
15,000–49,999 (n = 33)	11.0 (1.1)		99.9 (16.9)	
≥50,000 (n = 20)	13.2 (1.3)		122.1 (19.8)	
Not reported (n = 5)	12.4 (2.1)		91.2 (32.5)	

Adjusting for all variables listed in the table

*SE* standard error

As observed in both the unadjusted (Table 2) and adjusted (Table 3) results, parents born outside the U.S. generally had longer family meals than those born in the U.S. As shown in Table 4, this difference in mean duration is not only modified by the parents’ race/ethnicity (*p* = 0.45 for test of interaction between race/ethnicity and born in the U.S.) but there appears to be more variability among the race/ethnicity groups in those born outside the U.S. (*p* = 0.14 for test of mean group differences) than in those born in the U.S. (*p* = 0.43 for test of mean group differences). The average time spent per day eating family meals ranged from 63.7 min among multi-racial/other parents born in the U.S. to 182.8 min among black parents born outside of the U.S.

**Table 4:** Comparison of adjusted mean number of family meals per week and adjusted mean number of minutes in family meals per day by race/ethnicity and born in the U.S. from an analysis of covariance

Race/ethnicity	Mean number of meals per week (SE)		Mean number of minutes per day (SE)	
	Born in the U.S.		Born in the U.S.	
	Yes	No	Yes	No
White	13.0 (1.8)	13.7 (2.2)	71.1 (26.9)	134.4 (33.8)
Black	10.4 (1.2)	13.8 (1.8)	102.6 (18.3)	182.8 (26.6)
Hispanic	12.2 (2.1)	11.9 (1.3)	104.1 (31.3)	119.6 (20.2)
Multi-racial/other	11.8 (1.6)	15.1 (3.2)	63.7 (24.5)	124.8 (49.0)
*p* value	0.48	0.56	0.43	0.14

Adjusting for all variables listed in the Table 3

*SE* standard error

## Discussion

In this sample of parents of third- through sixth-grade students, all but one parent ate at least one meal with their child per week. We found that sociodemographic characteristics were not significantly associated with the frequency with which parents ate meals with their children per week. However, we did find associations with sociodemographic characteristics in the average duration parents spent eating meals with their children per day. Parents who were single, divorced, or separated, on average, spent significantly more time per day eating meals with their child than married or partnered parents. This finding is inconsistent with our hypothesis that parents who were not partnered would spend less time eating family meals, as well as with research outside of the U.S. indicating that single parents spend less time eating meals with their children (Raymo et al. 2014). It may be that these parents are more attuned to the needs of spending time with their children; however, more research is warranted to fully understand this relationship in the U.S. Additionally, those living in homes with more than one caregiver—who may a spouse, or a child’s grandparent or aunt/uncle—also spent more time eating meals with their child, which may be related to having an extra person in the home to help prioritize or prepare meals.

Additionally, there were differences in duration of meals by parental nativity and race/ethnicity, where, for example, those who were born outside the U.S. who identified as white ate together with their child for approximately one hour more on average than those who identified as white and were born in the U.S. This is consistent with literature indicating that people from other countries tend to spend more time eating in general and with their families than those born in the U.S., as mealtime is valued more (Boyle and Long 2013; Trends 2013). In the current sample, those who emigrated from other countries retained this practice within their families while living in the U.S. This cultural value may be an important protective factor for these families, as their children become adolescents. Additionally, socioeconomic status may play a role in these associations, which was not addressed in this study. Future research should determine whether time of emigration—as children or adults—is differentially related to time spend in family meals, and how socioeconomic status may impact these associations.

A strength of the study was that the sample was very similar to the population from which it was drawn with respect to race/ethnicity and income distribution from five public schools in a large city. Additionally, to the best of our knowledge, this analysis is the first to investigate associations with both frequency and duration of meals among a U.S.-based sample. The current study has some limitations that are important to note. First, this was a relatively small convenience sample, which limited our ability to declare apparent associations and interactions as statistically significant. Second, measures were self-reported, increasing the possibility of self-report bias. Finally, we did not assess whether parents and children had conversations during mealtimes, and distractions could have been present (e.g., television) that limited mealtime interactions.

## Conclusions

The results of this study provide a better understanding of which families are more likely to eat meals together and for longer periods of time. Future epidemiologic research with larger samples would help to better characterize families who eat together more often and for longer durations and incorporate measurements of behavioral outcomes of children as a way to delineate targets for prevention of future maladaptive behaviors.
